# Overexpression of a New Chitinase Gene *EuCHIT2* Enhances Resistance to *Erysiphe cichoracearum* DC. in Tobacco Plants

**DOI:** 10.3390/ijms18112361

**Published:** 2017-11-07

**Authors:** Xuan Dong, Yichen Zhao, Xin Ran, Linxia Guo, De-Gang Zhao

**Affiliations:** 1 The Key Laboratory of Plant Resources Conservation and Germplasm Innovation in Mountainous Region (Ministry of Education), Institute of Agro-Bioengineering and College of Life Sciences, Guizhou University, Guiyang 550025, China; xdong4501@gmail.com (X.D.); yczhao@gzu.edu.cn (Y.Z.); ranxin1024@gmail.com (X.R.); l.zoubingjie@gmail.com (L.G.); 2 The State Key Laboratory Breeding Base of Green Pesticide and Agricultural Bioengineering, Guizhou University, Guiyang 550025, China; 3 The School of Pharmaceutical Science, Guizhou University, Guiyang 550025, China; 4 Guizhou Academy of Agricultural Science, Guiyang 550025, China

**Keywords:** *Eucommia ulmoides*, chitinase, *Erysiphe cichoracearum* DC., transgenic tobacco

## Abstract

In this study, we cloned a new chitinase gene, *EuCHIT2*, from *Eucommia ulmoides* Oliver (*E. ulmoides*) using rapid amplification of cDNA ends (RACE) technology and constructed an overexpression vector, pSH-35S-*EuCHIT2*, to introduce it into tobacco (*Nicotiana tabacum* cv. Xanthi). Resistance to *Erysiphe cichoracearum* de Candolle (*E. cichoracearum* DC.) and molecular mechanisms in the transgenic tobacco were determined by drop inoculation, spore counting, determination of physicochemical indicators, and analysis of gene expression. The chitinase activity and resistance to *E. cichoracearum* DC. were significantly higher in the transgenic tobacco than in wild-type tobacco (*p* < 0.05). The activities of peroxidase (POD) and catalase (CAT), after inoculation with *E. cichoracearum* DC., were higher in the transgenic tobacco than in the wild-type. Conversely, the malondialdehyde (MDA) content was significantly lower in the transgenic tobacco than the wild-type before and after inoculation. In addition, our study also indicated that the resistance to *E. cichoracearum* DC. might involve the salicylic acid (SA) and jasmonic acid (JA) pathways, because the expression levels of pathogenesis-related gene 1 (*PR-1a*) and *coronatine-insensitive 1* (*COI1*) were significantly increased and decreased, respectively, after inoculation with *E. cichoracearum* DC. The present study supports the notion that *PR-1a* and POD participate in resistance to *E. cichoracearum* DC. in the transgenic tobacco plants.

## 1. Introduction

*Eucommia ulmoides* Oliver (*E. ulmoides*), a living fossil and the only surviving species in both its genus and family, is a deciduous tree, which is also one of the few tertiary species that have survived only in China. The bark and leaf of *E. ulmoides* are traditional Chinese medicines [1] and were recorded as being used as medicine in “Shennong’s Herbal Classic”, the earliest medicinal plant bibliography in ancient China. The medicinal value of *E. ulmoides* is mainly through the two-way regulation of blood pressure and bacteriostasis [2]. In 2002, Huang et al. [3] purified two novel antifungal peptides, EAFP1 and EAFP2, from the bark of *E. ulmoides*, which showed a relatively broad spectra of antifungal activities against eight pathogenic fungi. They also found that EAFP1 and EAFP2 revealed characteristics of the hevein domain and had chitin-binding properties similar to hevein-like peptides. Both of these two proteins could provide resistance to fungi with or without chitin contained in the cell walls [3]. Hevein is one of the major proteins in the lutoid bodies of rubber tree latex, which bind chitin and inhibit the growth of several chitin-containing fungi [4,5]. It is known that *E. ulmoides* can possess a kind of latex similar to that of *Hevea brasiliensis* (*H. brasiliensis*, the structure of *E. ulmoides* rubber is trans-polyisoprene, while *H. brasiliensis* rubber is *cis*-polyisoprene) [4,5,6]. Our research group has conducted the study of the antifungal activity of *E. ulmoides* for many years and isolated a novel antifungal peptide, EAFP3, from its bark in 2008 [7,8,9].

Chitinases (EC 3.2.1.14) catalyze the hydrolytic cleavage of the β-1,4-glycoside bond of *N*-acetylglucosamine (GlcNAc), the principal monosaccharide in chitin. Chitinases are present in various organisms and are considered to be important pathogenesis-related (PR) proteins involved in plant immune systems [10,11,12]. Exogenous application of salicylic acid, the key phytohormone of plant systemic acquired resistance (SAR), or jasmonic acid, the key phytohormone to plant-induced systemic resistance (ISR), can induce expression of protein PR-3 (chitinase) or PR-4 (chitinase), respectively [13,14,15]. Hence, understanding exactly how chitinases are involved in plant immune systems and what kind of roles they play is important for the study of plant resistance.

Powdery mildew is estimated to cause 30% to 80% damages and losses in tobacco growth worldwide [16]. Guizhou is the main tobacco-growing province in China, where the humid climate favors the development of powdery mildew. Chemical fungicides are commonly used and are efficient for controlling *Erysiphe cichoracearum* de Candolle (*E. cichoracearum* DC.) in tobacco; however, they may cause unforeseen and devastating side effects on the environment. Nowadays, in order to reduce the use of chemical fungicides increasing the plants, SAR and ISR via transgene technology have attracted more and more attention [17,18,19].

Based on previous studies of transcriptomics and antifungal proteins of *E. ulmoides*, we assume that chitinase is involved in the antimicrobial activity in *E. ulmoides*. Thus, in order to find evidence to support the hypothesis, a plant overexpression vector containing a chtinase gene *EuCHIT2*, the novel chitinase gene cloned in this study, was constructed and introduced into the tobacco plant.

## 2. Results

### 2.1. Cloning and Characterization of EuCHIT2

**Figure 1:**
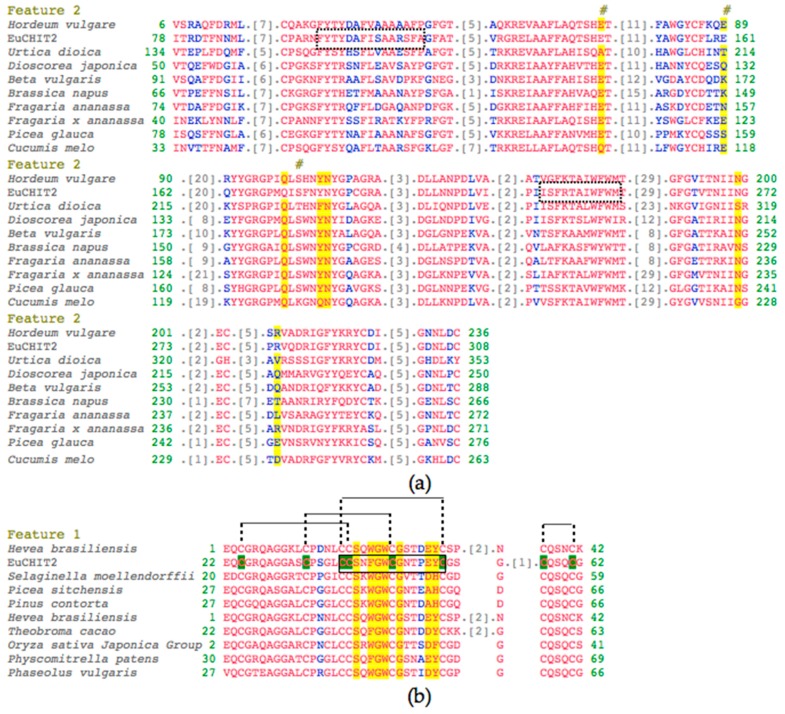
Alignment analysis of EuCHIT2 with other organisms. (**a**) Alignment of deduced amino acid sequence of EuCHIT2 with family 19 chitinases different organisms. The two chitinase family 19 signature sequences FYTYDAFISAARSFAGFA (95–117 aa) and ISFRTAIWFWM (221–231 aa) were surrounded with imaginary frames; (**b**) alignment of deduced amino acid sequence of EuCHIT2 with ChtBD_CH19_hevein (a hevein protein) of other organisms. The chitin recognition domain signature sequence CCSNFGWCGNTPEYC was surrounded with black frame and the eight “C” residues highlighted in green are the conserved cysteine residues that can form four disulfide bonds in the chitin-binding region of EuCHIT2. The species shown in the figure are: 2BAA (*Hordeum vulgare*), P11218.3 (*Urtica dioica*), P80052.2 (*Dioscorea japonica*), and P42820.1 (*Beta vulgaris*); and Q43391 (*Brassica napus*), AAD28733.1 (*Triticum aestivum*), AAF00131.1 (*Fragaria* x *ananassa*), Q40838 (*Picea glauca*), and AAF64475.1 (*Cucumis melo*); and 1WKX_A (*Hevea brasiliensis*), EFJ22468.1 (*Selaginella moellendorffii*), ABK24218.1 (*Picea sitchensis*), AEF59005.1 (*Pinus contorta*), 1Q9B_A (*Hevea brasiliensis*), Q41596.1 (*Theobroma cacao*), 2DKV_A (*Oryza sativa Japonica* Group), XP_001767856.1 (*Physcomitrella patens*), and CAB97002.1 (*Phaseolus vulgaris*)*.* The red and blue letters are residues in the chitinase domain share high and relatively higher similarity, respectively. The grey number represents the number of amino acid residues that have been omitted. The amino acid residues highlighted in yellow are sugar-binding residues, and the residues under the # markers are catalytic residues.

The full-length cDNA of *EuCHIT2* was obtained from the total RNA of *E. ulmoides* by rapid amplification of cDNA Ends (RACE) technology and Polymerase Chain Reaction (PCR) method, based on a 276 bp contig partial chitinase gene fragment that was annotated in the *E. ulmoides* transcriptome. Sequence analysis indicated that *EuCHIT2* was 1218 bp in length with an open reading frame (ORF) of 972 bp, which encoded a 324 amino acid (aa) protein with a theoretical molecular weight of 34.7 KDa and an isoelectric point of 4.68. The *EuCHIT2* cDNA sequence has been deposited in GenBank with accession number KJ413009.1 (Figure S1). The deduced amino acid of EuCHIT2 was aligned against the chitinase sequences from other organisms in NCBI (Basic Local Alignment Search Tool), which revealed eight residues that were well conserved, namely, the catalytic residues 139(E), 161(E), and192(S), the sugar-binding residues 190(Q), 195(Y), 196(N), 271(N), and 283(R) (Figure 1a and Figure S2) of chitinase 19 family. We also found a hevein or type 1 chitin-binding domain in the region 22–62. Alignment of the deduced amino acid sequence against hevein sequences from other organisms showed that the seven residues in the carbohydrate-binding site were quite well-conserved, namely, 40(N), 42(F), 43(G), 44(W), 46(G), 50(E), 51(Y), and 46(G) (Figure 1b). The motif analysis from ScanProsite (http://prosite.expasy.org/scanprosite/) predicted that there was a conserved chitin-binding region at position 21–63 aa in the EuCHIT2 sequence, which contained eight cysteine residues that formed four disulfide bonds. This indicated that EuCHIT2 is a typical chitinase with one chitin-binding sequence CCSNFGWCGNTPEYC (33–52 aa) and two chitinase family 19 signature sequences, FYTYDAFISAARSFAGFA (95–117 aa) and ISFRTAIWFWM (221–231 aa) (Figure 1). We also used SignalP 4.1 (Available online: http://www.cbs.dtu.dk) to predict the EuCHIT2 protein and found it may contain eight serine phosphorylation sites, six threonine phosphorylation sites, and three tyrosine phosphorylation sites, and that there was a secretory pathway signal peptide outside the membrane.

### 2.2. Phylogenetic Analysis

**Figure 2:**
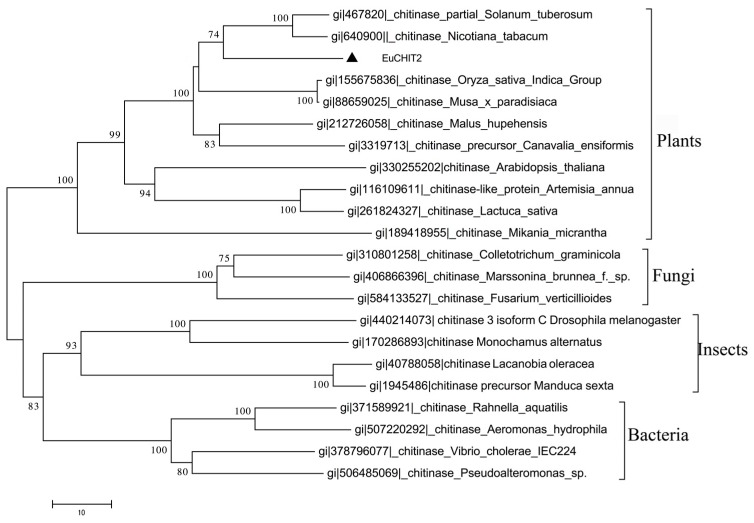
Phylogenetic analyze on the alignment of deduced EuCHIT2 amino acid sequences with chitinase from plants, bacteria, fungi, and insects. EuCHIT2 formed a cluster with the chitinases origin from plants and shares a closer relationship with the chitinases of *S. tuberosum* and *N. tabacum*.

The results of phylogenetic analysis are shown in Figure 2, which reveals that EuCHIT2 clustered with the plant chitinase group and had a closer relationship with *N. tabacum*. Meanwhile, the result of phylogenetic analysis suggested that all chitinases evolved from the same ancestor, and that EuCHIT2 shared a common evolutionary origin with chitinases from other plants (Figure 2).

### 2.3. Verification of Vectors and Transgenic Tobacco Plants

A plant overexpression vector pSH-35s-*EuCHIT2* was constructed, which included the 35S promoter-induced *EuCHIT2* sequence. The pSH-35s-*EuCHIT2* was transformed into tobacco plant and the positive transgenic plants were selected using GUS staining and PCR amplification (Figures S3 and S4). The results show that a total of 36 transgenic lines were regenerated from the 150 resistant tobacco seedings, and the transformation efficiency was about 24%. Three lines with the best growing status and sufficient seeds were chosen for the following experiments.

### 2.4. Chitinase Activity in the Transgenic Tobacco Lines

**Figure 3:**
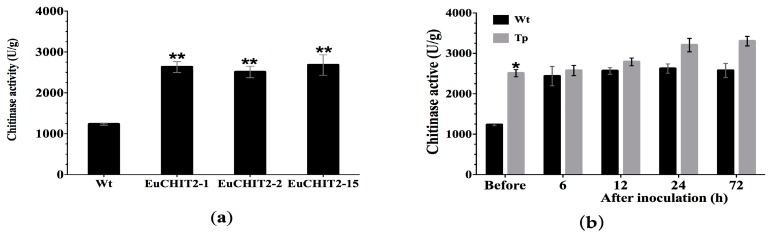
Chitinase activities of wild-type and transgenic tobacco plants challenged with *E. cichoracearum* de Candolle. (**a**) Chitinase activities in uninoculatie plant (*p* < 0.01); (**b**) chitinase activities of T1 EuCHIT2 transgenic lines before inoculation and 6, 12, 24, and 72 h after inoculation with *E. cichoracearum* DC. Wt, wild-type, (*n* = 3); Tp, transgenic plant (EuCHIT2-15), (*n* = 3). Error bars indicate standard error (SE); * and ** indicate significant differences at *p* < 0.05 and *p* < 0.01, respectively.

The chitinase activity was significantly higher in the three different transgenic tobacco lines (EuCHIT2-1, EuCHIT2-2, and EuCHIT2-15) than in the wild-type before *E. cichoracearum* DC. inoculation (Figure 3a). However, there was no signification difference in the chitinase activity between the wild-type and transgenic tobacco lines after inoculation (Figure 3b).

### 2.5. Disease Resistance Assay with E. cichoracearum DC.

**Figure 4:**
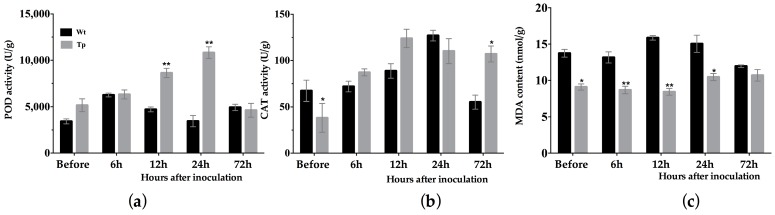
Determination of physicochemical indexes in *E. cichoracearum* DC.-inoculated, transgenic (*n* = 3) and wild-type (*n* = 3) tobacco plants. (**a**) Peroxidase (POD) activity; (**b**) catalase (CAT) activity; (**c**) malondialdehyde (MDA) content. Wt, wild-type; Tp, transgenic plant. Error bars indicate SE, and * and ** indicate significant differences at *p* < 0.05 and *p* < 0.01, respectively.

In this study, the peroxidase (POD) activity in the transgenic tobacco was significantly higher than in wild-type 6, 12, and 24 h before and after inoculation, respectively. However, at other time points after inoculation, no significant differences were detected between transgenic and wild-type tobacco (Figure 4a). The catalase (CAT) activity in the wild-type tobacco was 1.76 times higher than that in the transgenic tobaccos before inoculation. However, 72 h after inoculation, the CAT activity in the transgenic tobaccos was 1.94 times higher than that in the wild-type tobacco (Figure 4b). The malondialdehyde (MDA) content before and 6, 12, and 24 h after inoculation was significantly lower in the transgenic tobacco than in the wild-type tobacco (Figure 4c)

**Figure 5:**
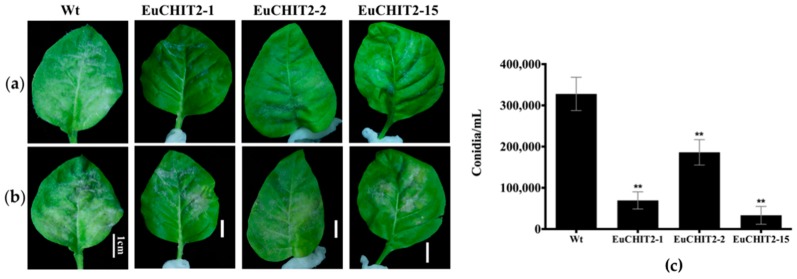
Disease development on *N. tabacum* cv. Xanthi leaves with *E. cichoracearum* DC. inoculation. (**a**) 7 days after inoculation, and (**b**) 12 days after inoculation. Wt, wild-type, Bars = 1 cm; (**c**) mean number of conidia on leaves of three individual transgenic lines (*n* = 9 in each) and the wild-type tobacco plants (*n* = 9) 12 days after inoculation with *E. cichoracearum* DC. Error bars indicate standard deviation, and ** indicates significant differences at *p* < 0.01, respectively.

A small lesion appeared 3 days after inoculation in the wild-type and the transgenic tobacco line EuCHIT2-2, but the symptoms were more severe in the wild-type than in the transgenic tobacco. At 12 days after inoculation, the *E. cichoracearum* DC. mycelium and spores were dispersed over the leaf surface in the wild-type tobacco; although all the transgenic tobacco shows the same symptoms, they were not as severe as the wild-type tobacco symptoms. Spore counting was performed at 12 days after inoculation, and the average numbers of spores grown on the wild-type were 4.72-fold, 1.76-fold, and 9.83-fold higher than the infected leaves from EuCHIT2-1, EuCHIT2-2, and EuCHIT2-15, respectively. Moreover, the number of conidia of *E. cichoracearum* DC. on the transgenic tobacco leaves was significantly lower than on the wild-type tobacco leaves. This result indicates that overexpression of the *EuCHIT2* transgene in tobacco could increase resistance to *E. cichoracearum* DC. infection (Figure 5).

### 2.6. The Synergistic Effects of EuCHIT2 on E. cichoracearum DC. Resistance-Related Genes Expression

**Figure 6:**
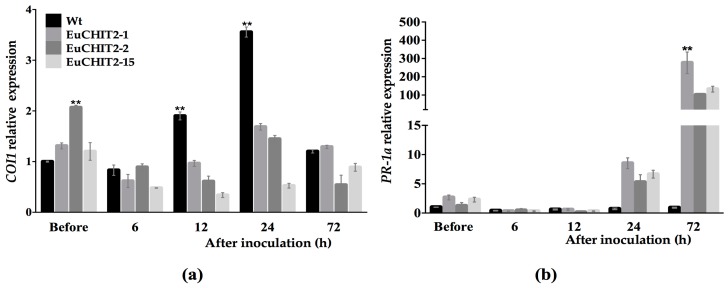
Relative expression levels of pathogen-related genes in Wt (wild-type) and transgenic tobacco leaves before and after of *E. cichoracearum* DC. inoculation, (*n* = 3). (**a**) *COI1* relative expression levels; (**b**) *PR-1a* relative expression levels. EuCHIT2-1, EuCHIT2-2, and EuCHIT2-15 are three individual transgenic tobacco lines with EuCHIT2 overexpression. Error bars indicate standard deviation, and ** indicates significant differences at *p* < 0.01, respectively.

Pathogenesis-related 1a (*PR-1a*) and coronatine-insensitive 1 (*COI1*) are two pathogen-related genes involved in plant SAR and ISR, respectively. The results show that before inoculation, *COI1* expression was 1.53 times higher in the transgenic tobacco than in the wild-type. However, at 24 h after inoculation with *E. cichoracearum* DC., *COI1* was 2.92 times higher in the wild-type tobacco than in the transgenic tobacco. At 24 and 72 h after inoculation, *PR-1a* expression was 9.46 and 186.82 times higher in the transgenic tobacco than in the wild-type tobacco, respectively. These results suggest that when the *EuCHIT2* gene is introduced into tobacco, it stimulates resistance to *E. cichoracearum* DC. by inducing the expression of *PR-1a* (Figure 6).

## 3. Discussion

Accumulating evidence has indicated that plant chitinases are important PR proteins that play a major role in plant defense systems [20]. Higher plants produce several chitinases that are encoded by a gene family, and they share high sequence similarities [21]. Studying the function of each member of this family is important for a better understanding of the defense system in plants. *E. ulmoides* is a valued rubber trees species and a traditional Chinese medicinal plant that has antifungal properties. Here, we cloned and analyzed a new chitinase gene from *E. ulmoides*. Analysis of the conserved regions of this protein shows that it belonged to the chitinase glycoside hydrolase 19 family and contained the catalytic and sugar-binding residues that are conserved in this family. Besides this, an additional carbohydrate binding site was found in the *N*-terminal region of this protein, which belonged to the ChtBD1-GH19-hevein subfamily. The subfamily contains two important domains: one is the hevein protein domain, which is a major IgE-binding allergen in natural rubber latex; the other is the ChTBD1 domain, a lectin domain [22,23,24]. ChTBD1 is involved in the recognition and binding of chitin subunits and typically occurs at the *N*-terminal of the glycosyl hydrolase domains in chitinases [25], together with other carbohydrate-binding domains, or by itself in a tandem-repeat arrangement [26,27]. In 1990, Broekaert et al. found that wounding could induce the transcription of the hevein in laticifers of rubber trees (*H. brasiliensis*) [4,28]. This discovery linked wounding with rubber production. In *H. brasiliensis*, latex is produced from specific cells called laticifers and plays an important role in sealing the wound, which prevents pathogens from entering the phloem [29]. Hevein is one of the major proteins in rubber tree latex [30]. *E. ulmoides* is a cultivated rubber species in China, and in all rubber tissues of *E. ulmoides*, the filament single cells are filled with rubber particles [31,32]. Therefore, it may be worth investigating whether or not *Eucommia* rubber has antimicrobial activity.

When pathogenic microorganisms invade plants and stimulate the plant immune system, a hypersensitive response is activated at the infected site to inhibit the spread of the microorganism. This process can lead to the accumulation of ROS [33,34] and the activation of plant defense enzymes that help to maintain cell integrity and remove the peroxide, or that have a direct effect on the pathogenic microorganisms. In this study, POD activity was higher in uninfected plants and in early infection with *E. cichoracearum* DC. in the transgenic tobacco than in the wild-type. Moreover, CAT activity was also significantly higher in the transgenic tobacco than in the wild-type 72 h after inoculation. The MDA content was lower in the transgenic tobacco than in the wild-type (Figure 4). These results indicate that overexpression of the *EuCHIT2* in tobacco increases the ability of POD and CAT to maintain cell integrity in response to *E. cichoracearum* DC. infection.

Generally, plant chitinases can be induced in response to various pathogenic microorganisms [35,36,37,38]. In this study, we found that the chitinase activity was significantly higher in the transgenic tobacco than in the wild-type before inoculation. However, after inoculation with *E. cichoracearum* DC., there was no difference in the chitinase activity in transgenic and the wild-type tobacco (Figure 3). This result indicates that besides antifungal activity, EuCHIT2 also serves as an activator to trigger or interact with other defense-related proteins. Thus, chitinase may be involved in both direct and indirect defense reactions against *E. cichoracearum* DC. Plants have a broad spectrum of resistance to pathogens and can respond to infection caused by many pathogenic organisms. SAR and ISR are two types of plant defense systems. These two defense systems are derived from previous infections or previous pretreatments and confer resistance to a subsequent challenge by a pathogen. *COI1* plays an important role in the pathway of ISR that is dependent on jasmonic acid [39,40], and PR-1a is the marker protein in the pathway of SAR that is dependent on salicylic acid [41]. In our study, *COI1* expression was higher in the transgenic tobacco before inoculation, implying that overexpression of *EuCHIT2* in tobacco may affect the ISR pathway to increase *COI1* expression. However, PR-1a expression was significantly higher in the transgenic tobacco at 72 h after inoculation, implying that the reaction between EuCHIT2 and *E. cichoracearum* DC. dramatically increased *PR-1a* expression. This result indicates that the pathogenesis-related protein PR-1a may play a role in the resistance mechanism against *E. cichoracearum* DC. in a EuCHIT2-mediated, disease-resistant signaling pathway. After all, current results suggest that overexpression of EuCHIT2 in tobacco plants could increase the resistance against *E. cichoracearum* DC., which offers us some circumstantial evidence that EuCHIT2 could relate to antifungal activity in *E. ulmoides*. However, the mechanism of this gene and its role involved in *E. ulmoides* resistance is still unknown and requires further research.

## 4. Materials and Methods

### 4.1. Materials

All the *E. ulmoides* were cultivated in the experimental field of the Key Laboratory of Plant Resources Conservation and Germplasm Innovation in the mountainous region of Guizhou University in Guiyang, China. Total RNA was extracted from young leaves and stems of mature *E. ulmoides*, *N. tabacum* cv. Xanthi, *E. coli* strain DH5α, and *A. tumefaciens* strain LBA4404, which were maintained by the same laboratory. The *E. cichoracearum* DC. spores were collected from detached leaves of greenhouse-grown tobacco plants and identified [42].

### 4.2. Extraction Total RNA

Total RNA for RACE was extracted from a maximum of 100 mg of leaves or young stems of *E. ulmoides* tree using an EZNA^®^ Plant RNA Kit (Omega Bio-Tek, Norcross, GA, USA), according to the manufacturer’s instructions, for difficult samples. The products were washed twice with ethanol to remove salt and dissolved in 40 μL Rnase-free water.

Total RNA for RT-PCR was extracted from a maximum of 100 mg of tobacco leaf tissue that was ground to powder in liquid nitrogen. Fruit-mate^TM^ for RNA Purification (Takara, Dalian, China) and RNAiso Plus (Takara, Dalian, China) were used to extract total RNA. All steps were carried out according to the manufacturer’s instructions. The products were dissolved in 40 μL Rnase-free water. All RNA samples were tested for 260/280 nm absorption and their quality was determined with 1.2% agarose gel electrophoresis with GelRed Nucleic AcidBiotiumInc. (Fremont, CA, USA) staining.

### 4.3. Rapid Amplification of cDNA Ends

Based on the partial sequence of *EuCHIT2* in the transcriptome data (Figure S1) [43], gene-specific primers, *EuCHIT2*-GSP1 and *EuCHIT2*-GSP2 (Table 1), were designed for cloning the 5′ and 3′ ends of *EuCHIT2* by RACE. First-strand cDNA was obtained from 5 μg total RNA with a SMARTer RACE cDNA Amplification Kit (Clontech Laboratories Inc. Mountain View, CA, USA). First-round PCR was performed with Advantage^®^ 2 Polymerase Mix (Clontech Laboratories Inc. Mountain View, CA, USA) in a 50 μL reaction mixture containing 34.5 μL PCR-grade water, 2.5 μL first-strand cDNA (50 ng/μL), 5.0 μL 10× Advantage 2 PCR buffer, 1.0 μL dNTP Mix (10 mM), 5.0 μL 10×Universal Primer A Mix (UPM), 1 μL Gene-Specific Primers (GSP) (10 μM), and 1.0 μL 50× Advantage^®^ 2 Polymerase Mix. The PCR procedures were all started with 5 cycles at 94 °C for 30 s and 72 °C for 3 min, followed by 5 cycles at 94 °C for 30 s, 70 °C for 30 s, and 72 °C for 3 min, with a final 20 cycles at 94 °C for 30 s, 68 °C for 30 s, and 72 °C for 3 min. The PCR products were run on 1.0% agarose/GelRed gel, and the corresponding DNA bands were purified with an EZNA Gel Extraction Kit (Omega Bio-Tek, Norcross, GA, USA). The product was connected with a pGEM-T Easy Vector (Promega Corporation, Madison, WI, USA), amplified in *E. coli* DH5α and sequenced. The putative 3′- and 5′-RACE cDNAs were aligned with the fragment from the transcriptome library using Serial Cloner 2.1 (version 2.6.1, Frank Perez, serialcloner@serialbasics.com) [44] to form a cDNA contig. Sequence analysis was performed using BioX software (version 1.5.1, Erik Lagercrantz, http://www.ebioinformatics.org) and Serial Cloner 2.1 to determine the putative 5′-UTR, open reading frame (ORF), and 3′-UTR. A pair of primers, *EuCHIT2*-*Xba*I-F and *EuCHIT2*-*Eco*RI-R, was designed to amplify the ORF of *EuCHIT2* (Figure S1 and Table 1). All primers were synthesized by Invitrogen Trading Co., Ltd. (Invitrogen, Shanghai, China).

### 4.4. Bioinformatics Analysis of EuCHIT2

Similarity searches were conducted using BLASTn and BLASTp (Available online: http://blast.ncbi.nlm.nih. gov/Blast.cgi) for the nucleotide and amino acid sequences of *EuCHIT2*, respectively. The ORF was analyzed, and the deduced amino acid sequence was predicted by Serial Cloner 2.6.1. The molecular weight, isoelectric point (pI), and instability index of EuCHIT2 were calculated with ExPASy Tool ProtParam (Available online: http://web.expasy.org/protparam/). Conserved domains in the deduced EuCHIT2 sequence were identified with the Conserved Domain Database (CDD) Tool CD-Search (Available online: https://www.ncbi.nlm.nih.gov/Structure/cdd/wrpsb.cgi) [45]. The motif was analyzed through the online bioinformatic software ScanProsite (Available online: http://prosite.expasy.org/scanprosite/). Meanwhile, we also use online website CBS (center for biological sequence analysis, Available online: http://www.cbs.dtu.dk) to predict the phosphorylation sites, transmembrane regions, and subcellular localization of EuCHIT2 protein. The amino acid sequences of EuCHIT2 and chitinases from other organisms, including plants, bacteria, fungi, and insects, were used to construct a phylogenetic tree using software MEGA5.22 with the neighbor-joining method with 1000 permutations [33].

### 4.5. Construction of Plant Overexpression Vector

All DNA manipulations were performed using protocols in the fourth edition of Molecular Cloning [43]. The elements of the binary plasmid pSH737 were the same as those of Qin et al. [46], which was stored in −80 °C freezer in the Institute of Agro-Bioengineering of Guizhou University. The ORF of *EuCHIT2* was amplified by PCR with primers that had added restriction sites for *Xba*I and *Eco*RI at the 5’-end. The PCR product was directionally joined to the plant expression vector pSH-35S through the restriction sites for *Xba*I and *Eco*RI, and flanking the two sites were a cauliflower mosaic virus (CaMV) 35S promoter and a nopaline synthase terminator. The plant overexpression vector pSH-35S-*EuCHIT2* was constructed and introduced into *A. tumefaciens* strain LBA4404 using a freeze-thaw transformation method [47,48].

### 4.6. Genetic Transformation and Identification of Transgenic Plants

Pest- and disease-free, wild-type tobacco leaves were used for genetic transformation [49]. The T0 generation-resistant tobacco seedlings were complete with well-developed roots and were transplanted according to Qin et al. [50]. Each pot contained about 5% active organic fertilizer (soybean cake) and one seedling. At the 6–8 leaf stage, we added the same amount of this fertilizer to each pot.

The transplanted *Kan*-resistant plants were further screened by histochemical staining with Gus [51], and the Gus-positive plants were further identified by genomic PCR. To distinguish the endogenous chitinase gene from the transgene, a pair of primers for verification was designed based on the T-DNA sequence of pSH-35s (one from a section of sequence of the 35S promoter, one from a section of located *EuCHIT2* cDNA). These verification primers were used to amplify the 778 bp fragment by PCR using 20 ng total isolated DNA as the template. The PCR conditions were as follows: 5 min at 95 °C for preheating, 30 cycles of 1 min at 95 °C for denaturation, 1 min at 60 °C for annealing, 2 min at 72 °C for synthesis, and 7 min at 72 °C for final extension. Gus and PCR positive, EuCHIT2-overexpressing transgenic lines were retained for further analyses.

### 4.7. Analysis of the Chitinase Activity

Chitinase activity assay was carried on T1 generation transgenic (EuCHIT2-1, EuCHIT2-2, and EuCHIT2-15) and wild-type tobacco plants. All transgenic plants that were used for analysis were positivly confirmed for EuCHIT2 overexpression. Full seeds of tobacco were selected and surface disinfected with 3% H_2_O_2_ for 30 min, washed several times with sterile distilled water, and then germinated on Murashige-Skoog (1962) medium (MS) (Phyto Technology Laboratories, Lenexa, KS, Canada) with 30 g/L sucrose and 7 g/L agarose until the size of the second true leaf was similar to that of the cotyledon. The tobacco seedings were transferred to 50 mL MS in 12 or 5.5 cm diameter glass culture pots. Seedings were grown at 25 °C with a 16 h light/8 h dark cycle until the 6–8 leaf stage. The leaf tips of seedlings at similar growth stages were selected for determination of chitinase activity according to the method of Zeng et al. [51]. Chitinase activity of the tobacco seedling was measured before inoculation and at 6, 12, 24, and 72 h after inoculation with *E. cichoracearum* DC. Briefly, 100 mg tobacco leaves were homogenized with liquid nitrogen and mixed with sodium acetate buffer (50 mM sodium acetate, 100 μM phenylmethanesulfonyl fluoride (PMSF), and pH 5.0 (Amresco LLC, Solon, OH 44139 USA) on ice for 1 h. The homogenate was centrifuged at 15,000× *g* for 15 min, and the supernatant was used as the crude enzyme preparation [52]. Colloidal chitin was prepared as the reaction substrate according to the method of Shimahara et al. (1988) [53]. One unit of chitinase activity was defined as the release of 1 μg GlcNAc (Sigma-Aldrich, St. Louis, MI, USA) in 1 h. The changes of absorption were measured at 540 nm to calculate the enzymatic activity. All experiments were repeated three times.

### 4.8. Analysis of Pathogen Resistance

To test the resistance of the transgenic tobacco to fungal infections, an in vivo test was performed with *E. cichoracearum* DC. Nine 5–6 cm long leaf pieces from similar leaves were collected from each line of tobacco for analysis of chitinase activity. Inoculations were performed in 90 mm Petri dishes with 15 mL (10 g/L) agar medium and a single leaf per dish [54]. The inoculation method was as described previously by Miclot et al. [54,55]. The conidia of *E. cichoracearum* DC. were eluted with 15 mL of sterile distilled water with one drop of Tween-20 (Sigma-Aldrich, St. Louis, MI, USA) from six severely infected tobacco leaves. A concentration of 10^6^ conidia/mL conidial suspension was used for inoculation. Ten 5 µL drops were dispensed onto the upper surface of each leaf and spread over the entire leaf surface using a glass spatula. Then, the leaf was allowed to dry at room temperature for 25 min. Inoculated leaves were maintained at 25 °C with a 16 h light/8 h dark cycle [56,57]. Two leaf discs (area in 1 cm^2^) were cutting from a single leaf 12 days after inoculation for spore counting. In order to indicate the severity of the disease symptos, each leaf disc group was washed with 2 mL of an isotonic solution. Each sample were suspended in 20 ul conidial suspension and counted with a Malassez (0.0025 mm^2^) hemocytometer three times.

For analysis of the CAT and POD activities, the MDA content, *PR-1a* and *COI1* gene expressions, and tobacco plants (6–8 leaf stage) were also inoculated with 100 μL of conidial suspension on two leaves of each plant. Plants inoculated with sterile, distilled water with one drop of Tween-20 were used as controls. All experiments were repeated three times.

### 4.9. Determination of Physicochemical Indicators

The activities of CAT, POD, and MDA content were measured separately using commercial chemical assay kits (Suzhou Comin Biotechnology Co., Ltd., Suzhou, China) according to the manufacturer’s instructions [58]. Uninoculated control leaves were collected before inoculation, and inoculated leaves were collected at 6, 12, 24, and 72 h after inoculation from both transgenic and wild-type tobaccos. Samples were stored at −80 °C until they were used for analysis [50].

### 4.10. Analysis of Gene Expression

The expression of two key genes, *PR-1a* and *COI1*, which are involved in the SA and JA pathways, were analyzed to gain a preliminary understanding of the overexpression of *EuCHIT2* and how it might affect the defense network in tobacco. The *COI1* sequence (AY428737.1) was obtained from GenBank and used to design the RT-PCR primers. β-Actin primers were used as the internal control, and the *PR-1a* primers from Qin et al. [50] were used as shown in (Table 1). The total RNA from transgenic and wild-type plants before and at 6, 12, 24, and 72 h after inoculation was extracted and reverse-transcribed into cDNA by Reverse Transcriptase M-MLV (RNase H−) (Takara, Dalian, China ). An SYBR Green I Dye Kit (Applied Biosystems Inc., Foster, CA, USA) was used for RT-PCR analysis. The relative expression levels of *COI1* and *PR-1a* were analyzed. The amplification cocktail was: template RNA 1 μg, Oligo(dT)12–18 Primer (50 μM) 1 μL, and RNase free ddH_2_O up to 6 μL at 70 °C for 10 min; it was then placed on ice for 3 min. Then, 5× M-MLV Buffer 2 μL, dNTP Mixture (10 mM) 0.5 μL, RNase Inhibitor (40 U/μL) 0.25 μL, RTase M-MLV (RNase H−)(200 U/μL) 0.25 μL, and RNase free dH_2_O was added up to 10 μL. The PCR cycles were 42 °C for 1 h and 70 °C for 15 min; they were then on ice for 3 min. The RT-PCR reaction system was Power SYBR Green PCR Master (2×) 10 μL, forward primer 1.0 μL, reverse primer 1.0 μL, cDNA 2 μL, and ddH_2_O 6 μL. All experiments were repeated three times. The reaction cycle was 40 cycles at 50 °C for 2 min, 95 °C for 10 min, 95 °C for 15 s, 60 °C for 1 min, the 95 °C for 15 s, 60 °C for 1 min, and 95 °C for 15 s.

### 4.11. Statistical Analysis

Statistical analyses were conducted with the IBM SPSS Statistics 20 software [57]. The standard differentiation (SD) was calcuated and presented with the data. Statistical significance was analyzed by Student’s *t*-test. Significance levels were set up at 0.95 [59].

## 5. Conclusions

In this study, we describe a novel chitinase gene (*EuCHIT2*) that was cloned from *E. ulmoides*. Overexpression of this gene increased the activities of chitinase, POD, and CAT in tobacco and increased the resistance to *E. cichoracearum* DC. Additionally, overexpression of *EuCHIT2* affected the expression of *PR-1a* and *COI1*, key genes involved in SAR and ISR. We will continue to study the function of this gene through transformation of *E. ulmoides* and prokaryotic expression.

## Figures and Tables

**Table 1:** Primers used in the full-length cloning and expression analysis of *EuCHIT2*.

Use	Name	Sequence (5′–3′)
RACE	*EuCHIT2-GSP1*	CAGGCCATAGCTAGTGCTGATGTTG
	*EuCHIT2-GSP2*	CCCGCCCGGAACTTCTACACCTACG
Full-length	*EuCHIT2-Xba*I-F	GCTCTAGAGCAAATGAGGTTTACCTTGTCCACTCTCC
	*EuCHIT2-Eco*RI-R	CGGAATTCCGGACTACTGGGCCTGAACTAAAAGCC
Verification	*psH-35s*-F	TCGTCAACATGGTGGAGCACGAC
	*TEuCHIT2*-R	CGAATCCGGCGAAAGATCTG
Internal control	*Actin*-F	TGGTTAAGGCTGGATTTGCT
	*Actin*-R	TGCATCCTTTGACCCATAC
Expression Analysis	*PR-1a*-F	ACAGCTCGTGCAGATGTAGGT
	*PR-1a*-R	GCTAGGTTTTCGCCGTATTG
	*COI1*-F	GTTGTAGCCAGTGAGGGAAATA
	*COI1*-R	TTGCCCAGCAAGAGAATAGTAG

## Supplementary Materials

Supplementary materials can be found at www.mdpi.com/1422-0067/18/11/2361/s1.

## Abbreviations

*E. ulmoides*	*Eucommia ulmoides* oliver
RACE	Rapid amplification of cDNA ends
*E. cichoracearum* DC.	*Erysiphe cichoracearum* DC.
POD	Peroxidase
MDA	Malondialdehyde
SA	Salicylic acid
JA	Jasmonic acid
*PR-1a*	Pathogenesis-related gene 1
*COI1*	*Coronatine-insensitive 1*
GlcNAc	*N*-acetylglucosamine
SAR	Systemic acquired resistance
ISR	Induce systemic resistance
ORF	Open reading frame
aa	Amino acid
