# Supplementary material for: Overexpression of a New Chitinase Gene EuCHIT2 Enhances Resistance to Erysiphe cichoracearum DC. in Tobacco Plants
Source: Int J Mol Sci. 2017 Nov 7;18(11):2361. doi: 10.3390/ijms18112361 (PMC5713330; doi:10.3390/ijms18112361)
Supplement: Supplementary file 1 [file ijms-18-02361-s001.pdf]

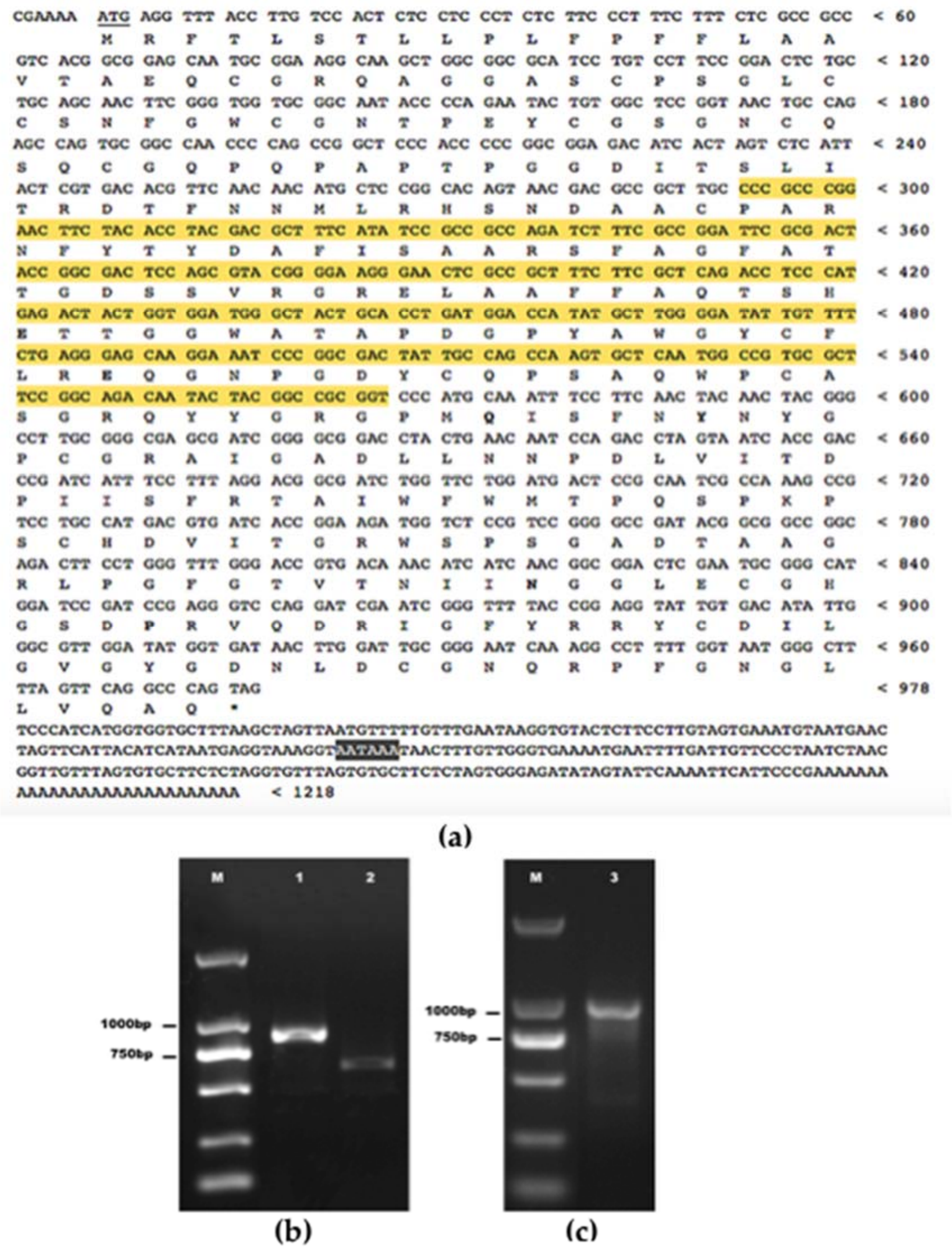

Supplementary Figure S1. RACE-PCR amplification and sequence analysis of *EuCHIT2*. (**a**) The nucleotide and deduced amino acid sequences of EuCHIT2. *EuCHIT2* (KJ413009.1) consists of 1218 bp with a 972-bp open reading frame encoding a protein with 324 amino acid residues. Start codon (ATG) is underlined; stop codon (TAG) is indicated by *. The amino acid residues critical for the chitinase catalytic activity are in bold font. The conserved AATAAA hexamer of the poly (A) signal is highlighted in grey. The overlapping RACE sequences are highlighted in yellow. **(b)** and **(c)** Electrophoresis of RACE-PCR products (**b**) Lane 1, EuCHIT2-3′ RACE (927 bp); lane 2, EuCHIT2-5′ RACE (567 bp); (**c**) lane 3, open reading frame of EuCHIT2; lane M, DL 2000 DNA marker. and

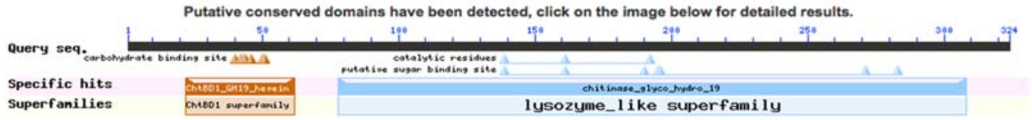

Supplementary Figure S2 Conserved domains of the deduced EuCHIT2 protein obtained using NCBI's Conserved Domain Database.

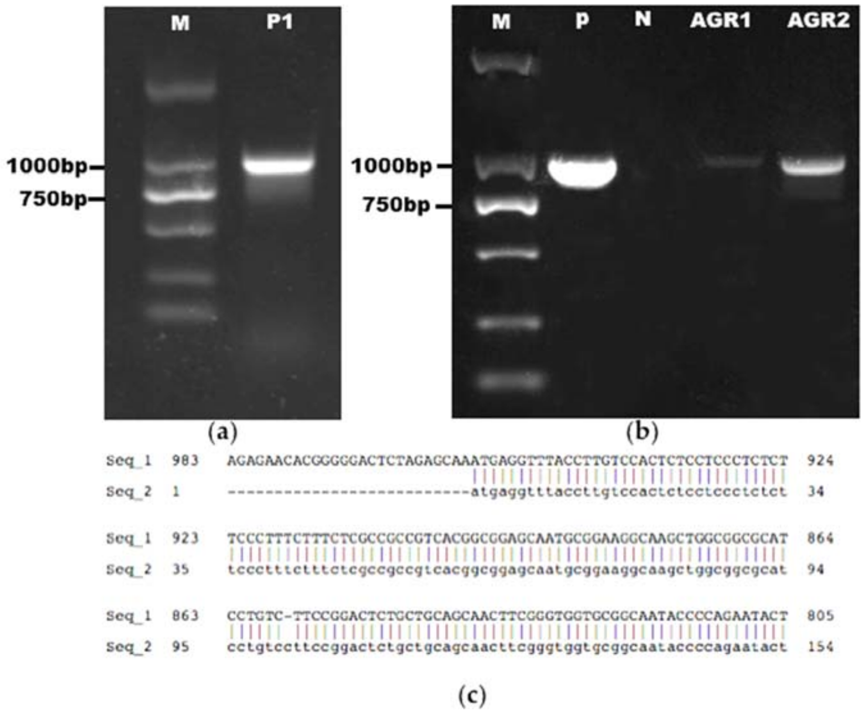

Supplementary Figure S3. Vector construction verification. A 996 bp fragment of full open reading frame was amplified with *EuCHIT2*-*Xba*I-F and *EuCHIT2*-*Eco*RI-R *EuCHIT2* primer. (a) Electrophoresis of PCR product amplified from plasmid vector pSH-*35s*-*EuCHIT2*. Full length of protein-coding region of *EuCHIT2* (996 bp) was shown in the gel image. Lane M, DL2000 DNA marker; lane P1, PCR amplification of plasmid vector pSH-*35s*-*EuCHIT2*. (b) Electrophoresis of PCR amplification for kanamycin-resistant Agrobacterium strains. A 996 bp lane was shown in the gel image. Lane M, DL 2000 DNA marker, lane P, positive control; lane N, negative control; lanes AGR1 and AGR2, kanamycin-resistance positive clones. (c) Sequences of the PCR amplification.

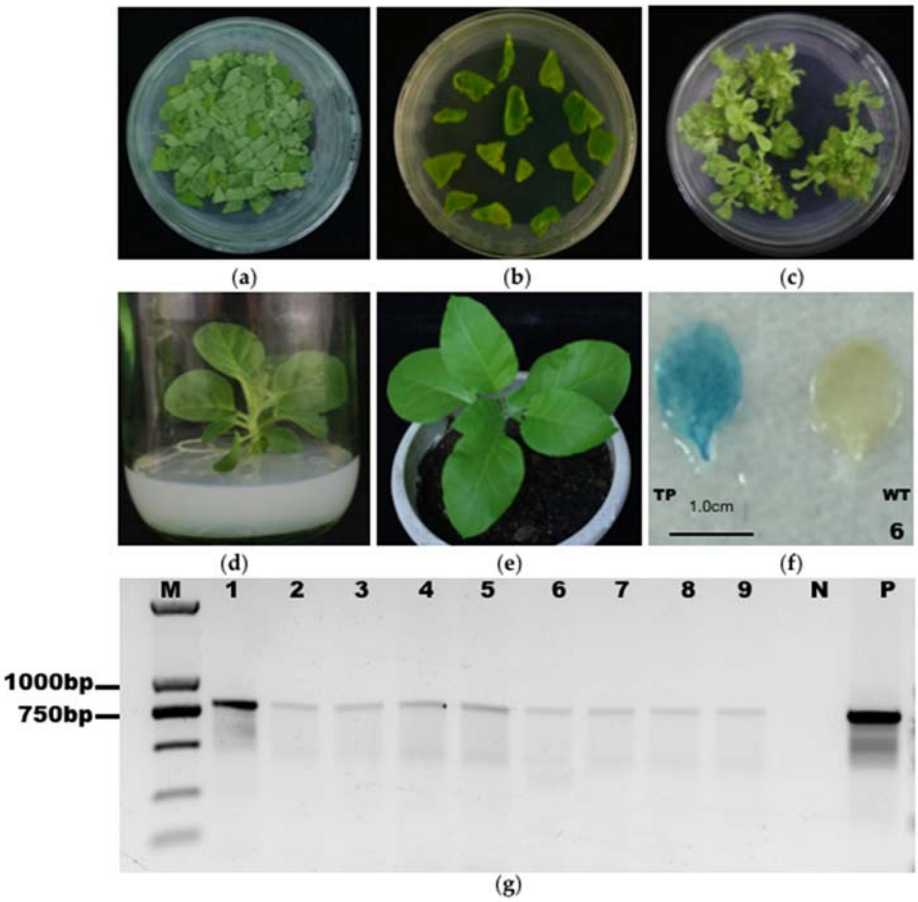

Supplementary Figure S4. Transformation and verification of transgenic tobacco plants. Tobacco was transformed with A. tumefaciens LBA4404 containing the overexpression vector pSH-35s-*EuCHIT2*, and co-cultured for 48 h. Transgenic tobacco plants generated with the EuCHIT2 overexpression vector were identified by Gus staining and PCR amplification by primers pSH-35s-F and TEuCHIT2-R to produce a 778 bp fragment that contained part of the 35S promoter fragment and part of the EuCHIT2 sequence (448 + 330 bp) (**a**–**e**) The transformation process in tobacco leaves. (**f**) Histochemical staining of the transgenic tobacco (TP) and wild type tobacco (WT) leaves. (**g**) Electrophoresis of PCR products. Lane M, DL2000 DNA marker; lanes 1–9, PCR amplification for Gus positives transgenic tobacco lines; lane N, wild type tobacco; lane P, plasmid pSH-35S-*EuCHIT*2.
